# The anti-tumor role of NK cells *in vivo* pre-activated and re-stimulated by interleukins in acute lymphoblastic leukemia

**DOI:** 10.18632/oncotarget.13007

**Published:** 2016-11-01

**Authors:** Fengyan Jin, Hai Lin, Sujun Gao, Zheng Hu, Song Zuo, Liguang Sun, Chunhui Jin, Wei Li, Yanping Yang

**Affiliations:** ^1^ Department of Hematology, The First Bethune Hospital of Jilin University, Changchun, China; ^2^ Institute of Translational Medicine, The First Bethune Hospital of Jilin University, Changchun, China

**Keywords:** natural killer cells, interleukin, interferon-γ, NKG2D, leukemia

## Abstract

Although natural killer cells (NK cells) were traditionally classified as members of the innate immune system, NK cells have recently been found also to be an important player in the adaptive immune systems. In this context, *in vitro* activation of NK cells by cytokines leads to generation of NK cells with memory-like properties characterized by increased interferon-γ (IFNγ) production. However, it remains to be defined whether these memory-like NK cells exist *in vivo* after cytokine activation. Furthermore, it is also unclear whether such memory-like NK cells induced *in vivo* by cytokines could have effective anti-leukemia response. To address these issues, we used an *in vivo* pre-activation and re-stimulation system that was able to produce NK cells with increased IFNγ secretion. It was found that after *in vivo* pre-activation and re-stimulation with interleukins (ILs), NK cells retained a state to produce increased amount of IFNγ. Of note, whereas this intrinsic capacity of enhanced IFNγ production after *in vivo* IL pre-activation and re-stimulation could be transferred to the next generation of NK cells and was associated with prolonged survival of the mice with acute lymphoid leukemia. Moreover, the anti-leukemia activity of these memory-like NK cells was associated with IFNγ production and up-regulation of NK cells activation receptor-NK Group 2 member D (NKG2D). Together, these findings argue strongly that *in vivo* IL pre-activation and re-stimulation is capable to induce memory-like NK cells as observed previously *in vitro*, which are effective against acute lymphoblastic leukemia, likely via NKG2D-dependent IFNγ production, in intact animals.

## INTRODUCTION

As NK cells play a crucial role in immunosurveillance against tumor formation, NK cells and their receptors can be targeted in many therapeutic approaches [1]. Although NK cells are traditionally considered to play the key role in the innate immune reaction, they have recently been discovered as an important component with memory-like function in the adaptive immune systems as well. Immunological memory is the ability of certain immune cells (e.g., T and B cells) to “remember” the first encounter with a pathogen and thus to provide an enhanced immune response to the secondary encounter with the same pathogen. Memory cells are often long-lived cells while phenotypically and epigenetically distinct from their naive counterparts, which thus respond more robustly to secondary infection than the latter. However, the classical concept of immunological memory solely applied to lymphocytes (i.e., T and B cells) has recently been challenged by the findings from the studies of NK cells, including that 1) antigen-specific NK memory cells can be induced by MCMV (mouse cytomegalovirus) infection; 2) NK memory cells can also be induced by exposure to cytokines alone; and 3) liver-restricted NK memory cells display highly antigen-specific recall immune responses [2]. In this context, activation of NK cells by cytokines alone leads to generation of NK cells with memory-like properties characterized by increased IFNγ production [3]. Notably, such a property of NK cells induced by cytokine itself are not found in the typical adaptive immune memory of B and T cells, as their activation requires both antigen-receptor signals and co-stimulation with cytokines [4]. Interestingly, the memory-like properties occur not only in NK cells that have not undergone cell division, but also in those that are the product of division [5]. The mechanism by which cytokine stimulation generates memory-like function of NK cells still remains unknown. However, there is a possibility that upon cytokine-induced activation, certain epigenetic changes occur at certain loci in NK cells, which imprint a memory-like phenotype. As NK cells constitutively express IFNγ transcripts [6], it is most likely that those epigenetic modifications appear at the loci responsible for post-transcriptional or post-translational up-regulation of this cytokine.

The NK cells activation receptor NKG2D is a C-type lectin-like activating receptor expressed on the surface of NK, CD8 T, and γδ T cells [7]. Its ligands (NKG2DL) are UL16-binding proteins, which are structurally similar to MHC class I molecules and thus comprise members of the MHC class I-related chain (MIC) family (MICA and MICB) [8, 9]. On the cell membrane, human NKG2D associates with DAP10, while mouse NKG2D may associate with both DAP10 and DAP12, which activate downstream signaling pathways, resulting in activation of NK cells as well as co-stimulation of CD8 T cells [10]. NKG2D-deficient mice are more prone to develop cancer than their wild-type counterparts [11]. Moreover, neutralization of NKG2D increases the susceptibility of mice to carcinogen-induced sarcomas [12]. Whereas NK cells secrete high levels of IFNγ in response to agonists of IL-12, this effect can be further enhanced by co-stimulation of NKG2D [13]. While the stimulatory effect of MICA-NKG2D interaction is able to induce NK cells with a capacity of IFNγ production [14], the number of NK cells secreting IFNγ is significantly reduced when NKG2D is blocked by anti-NKG2D antibodies [15].

It is noteworthy that virtually all of the earlier studies used the models of *in vitro* pre-activation and *ex vivo* or *in vivo* re-stimulation with cytokines. For example, in the study by Yokoyama et al., pre-activation by cytokines was carried out *in vitro*, followed by *ex vivo* re-stimulation for cytokine production [3]. However, after transfusion, NK cells are disabled early due to loss of IFNγ production, probably in association with down-regulation of the transcription factors Eomesodermin and T-bet [16]. Consequently, attempts so far to translate the promising biologic functions of NK cells *in vitro* activated by cytokines, through adoptive cell transfer (ACT), for the treatment of cancer have shown limited benefit. Therefore, certain critical issues remain to be addressed whether memory-like properties of NK cells also occur *in vivo* after activation with cytokines and whether such properties are required for anti-tumor activity of NK cells. To this end, a model of *in vivo* pre-activation and re-stimulation with cytokine was used in the present study. Here we report that NK cells indeed retained a state to produce increased amount of IFNγ state after *in vivo* interleukin (IL) pre-activation and re-stimulation. Such an intrinsic capacity of NK cells induced by *in vivo* IL pre-activation and re-stimulation not only could be passed to the next generation of NK cells, but also played an important role in anti-leukemia activity. Moreover, the mechanism underlying anti-leukemia activity of these NK cells was associated with increased IFNγ secretion via up-regulation of NKG2D. These findings indicate that the strategy of *in vivo* IL pre-activation and re-stimulation could induce retained memory-like NK cells with enhanced IFNγ production, which contribute to markedly increase anti-leukemia activity, thereby suggesting a novel and potentially effective approach of NK cell ACT therapy to treat acute lymphoblastic leukemia.

## RESULTS

### *In vivo* interleukin pre-activation and re-stimulation is able to induce memory-like NK cells with enhanced IFNγ production

**Figure 1:**
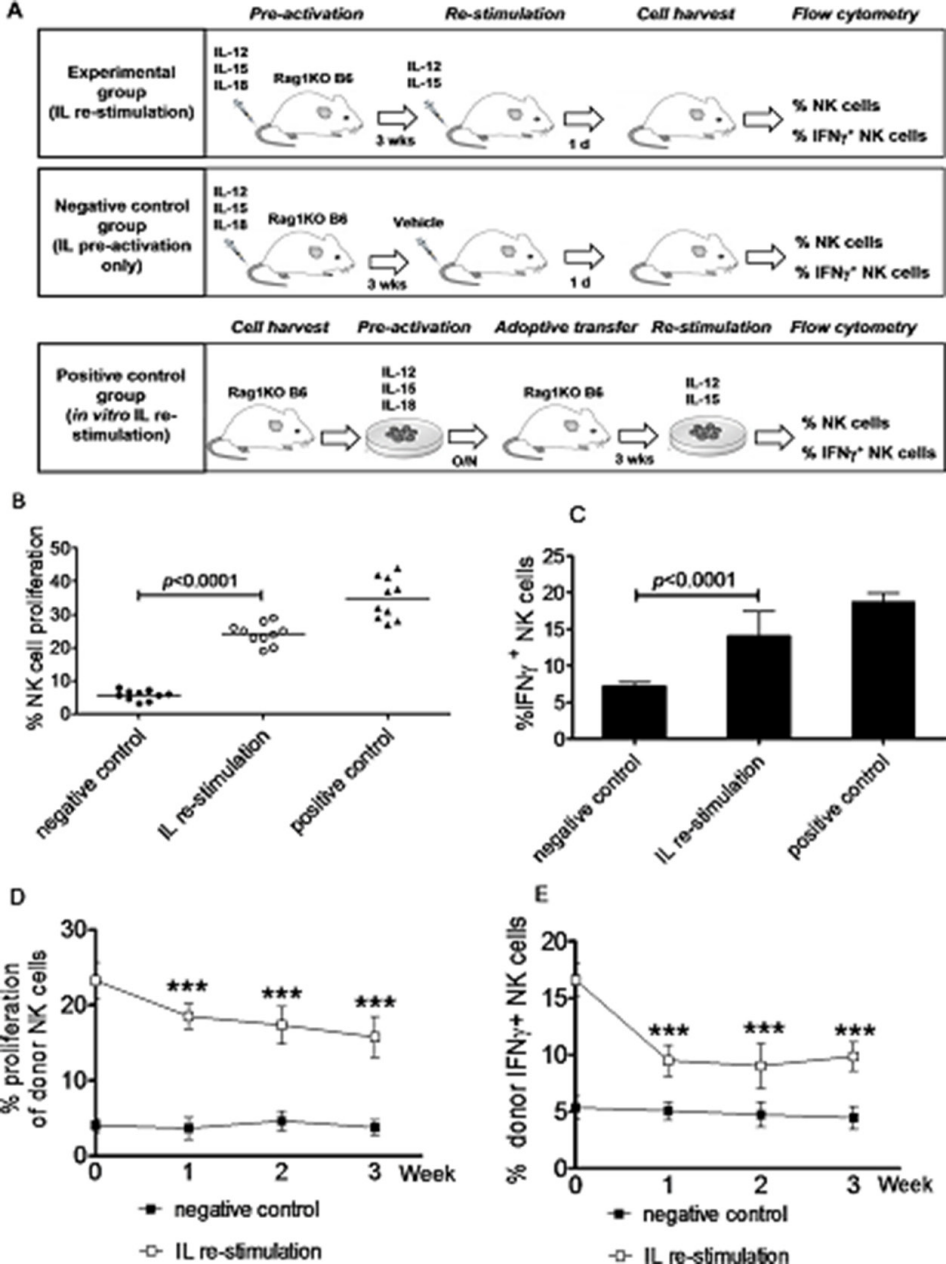
Generation of memory-like NK cells with enhanced IFNγ production by *in vivo* interleukin pre-activation and re-stimulation Rag1KO B6 mice were divided into three groups: (a) the IL re-stimulation group, in which mice received via tail vein a combination of IL-12 (4.5 μg/mouse), IL-15 (5.5 μg/mouse), and IL-18 (22 ng/mouse) for pre-activation, after three weeks, further received IL-12 (5 μg/mouse) and IL-15 (8 μg/mouse) for re-stimulation; (b) the negative control group, in which mice only received pre-activation as described above. For these two groups, NK cells were harvested from the spleen of the mice in next day after *in vivo* IL pre-activation and/or re-stimulation and subjected to further flow cytometric analyses; and (c) the positive control group, NK cells from the spleen of Rag1KO B6 donor mice were *in vitro* pre-activated with IL-12 (10 ng/mL), IL-15 (10 ng/mL), and IL-18 (50 ng/mL) overnight, after which NK cells were labeled with CFSE and adoptively transferred into Rag1KO B6 recipient mice. Three weeks later, enriched NK cells (2 × 10^6^) from spleen were harvested by negative selection and *in vitro* re-stimulated with IL-12 (10 ng/mL) and IL-15 (100 ng/mL) for 4 hrs, after which cells were further analyzed by flow cytometry. (**A**) Experiment schema for three groups. (**B**) The percentage of NK cell proliferation was analyzed by flow cytometry. (●) the negative control group; (○) the IL re-stimulation group; (▲) the positive control group. (**C**) The percentage of IFNγ^+^ NK cells in three groups. (**D**) Equal numbers (2 × 10^6^) of CFSE-labeled NK cells from the spleen of Rag1KO B6 (CD45.2+) mice pre-activated and/or re-stimulated with ILs as above were adoptively transferred to syngeneic B6 (CD45.1+) recipient mice, after which NK cells were harvested from peripheral blood of the recipients every week. Donor NK cells were distinguished from recipient NK cells using anti-A20 antibody. In A20^−^ donor NK cells, the percentage of NK cell proliferation were determined by flow cytometry at the indicated intervals after the adoptive infusion. (■) the negative control group; (□) the IL re-stimulation group. (**E**) In parallel, the percentage of IFNγ^+^ NK cells were also determined in A20^−^ donor NK cells. (■) the negative control group; (□) the IL re-stimulation group. Values indicate mean ± SD (*n* = 10 per group, *** *p* < 0.0001).

Memory-like NK cells that produce abundant IFNγ are virtually all generated by *in vitro* IL pre-activation [3]. Although these NK cells are able to traffic to tumor sites, they often, if not always, fail to control tumor growth or improve survival. Such dysfunction is associated with rapid down-regulation of activating receptor expression and loss of “effector” functions in these NK cells [16]. It has been reported that a population of MCMV-specific long-lived memory NK cells are able to respond robustly *in vivo* to subsequent challenge with MCMV [17]. Thus, we hypothesized that NK cells activated *in vivo* might be more effective, than NK cells activated *in vitro*, to treat leukemia. To address this hypothesis, we first attempted to generate memory-like NK cells with increased IFNγ production (IFNγ^+^ NK cells) using a new approach of *in vivo* IL stimulation for both pre-activation and re-stimulation. To this end, the proliferation rate of NK cells and the percentage of IFNγ^+^ NK cells after *in vivo* IL pre-activation and re-stimulation were first examined. Mice were randomly divided into three groups (Figure 1A), including the *in vivo* IL stimulation group, the negative-control group, and the positive-control group, in order to compare the number of NK cells and their capacity to produce IFNγ after IL pre-activation and re-stimulation in the different ways. In the *in vivo* IL stimulation group, mice received IL-12, IL-15, and IL-18 for pre-activation, followed by IL-12 and IL-15 for re-stimulation. In the negative-control group, mice received only pre-activation with IL-12, IL-15, and IL-18. In the positive-control group, NK cells isolated from the spleen of donor mice were pre-activated with IL-12, IL-15, and IL-18 for overnight, after which cells were labeled with carboxyfluorescein diacetate succinimidyl ester (CFSE) and then adoptively transferred into the recipient mice; three weeks later, enriched NK cells harvested from the spleen of the recipient mice were re-stimulated *in vitro* with IL-12 and IL-15. As shown in Figure 1 and Table 1, while the percentages of NK cells (24.23 ± 3.16%, Figure 1B) and IFNγ^+^ NK cells (14.09 ± 3.34%, Figure 1C) in the spleen of mice in the *in vivo* IL re-stimulation group did not reach the levels of NK cells in the positive-control group (NK, 34.87 ± 6.24%; IFNγ^+^ NK, 18.72 ± 3.97%), they were significantly increased, compared to those in the negative-control group (NK, 5.67 ± 1.52%; IFNγ^+^ NK, 7.22 ± 1.71%; *p* < 0.0001 for each case). Thus, although NK cells induced by *in vivo* IL pre-activation and re-stimulation displayed less proliferation and IFNγ production than those induced by *in vivo* pre-activation followed by *in vitro* re-stimulation with ILs, their capacity to proliferate and produce IFNγ was significantly stronger than the NK cells induced by *in vivo* IL pre-activation only.

We next determine whether NK cells induced by *in vivo* IL pre-activation and re-stimulation are able to maintain their capacity to secret increased amount of IFNγ after the syngeneic adoptive infusion in mice. After *in vivo* IL pre-activation with or without IL re-stimulation, equal numbers (2 × 10^6^) of NK cells isolated from the spleen of Rag1KO B6 (CD45.2+) mice were transferred to syngeneic B6 (CD45.1+) mice. After three weeks, NK cells harvested from the spleen of B6 (CD45.1+) mice were analyzed using specific anti-CD45.1 (A20) antibody by flow cytometry to distinguish the donor NK cells from those of the host, confirming the success of this ACT approach. On note, it was observed that the percentage of NK cells in the peripheral blood was significantly increased in the *in vivo* IL stimulation group, compared to those in the negative control group that only received IL pre-activation, at first (18.52 ± 1.69% vs 3.67 ± 0.84%, *p* < 0.0001), second (17.36 ± 2.46% vs 4.63 ± 0.75%, *p* < 0.0001), or third week (15.76 ± 2.71% vs 3.76 ± 0.59%, *p* < 0.0001) after the adoptive infusion (Figure 1D). Moreover, NK cells in the *in vivo* IL stimulation group produced the significantly increased amount of IFNγ, compared to those in the negative control group, at first (9.53 ± 1.36% vs 5.06 ± 0.79%, *p* < 0.0001), second (9.03 ± 1.96% vs 4.73 ± 1.09%, *p* < 0.0001), or third week (9.86 ± 1.27% vs 4.46 ± 0.97 %, *p* < 0.0001) post the adoptive infusion (Figure 1E). Together, the results indicate that *in vivo* IL pre-activation followed by re-stimulation is capable to produce NK cells with increased capacity of proliferation and IFNγ production. Importantly, although the half-life of NK cells is about 7 days [18], the present results also suggest that NK cells induced by this new approach could maintain their properties *in vivo* for at least three weeks after ACT, indicating long-lived memory-like cytokine responses.

### Continuous *in vivo* interleukin re-stimulation is not necessary for maintaining memory-like properties of NK cells with enhanced IFNγ production

**Figure 2:**
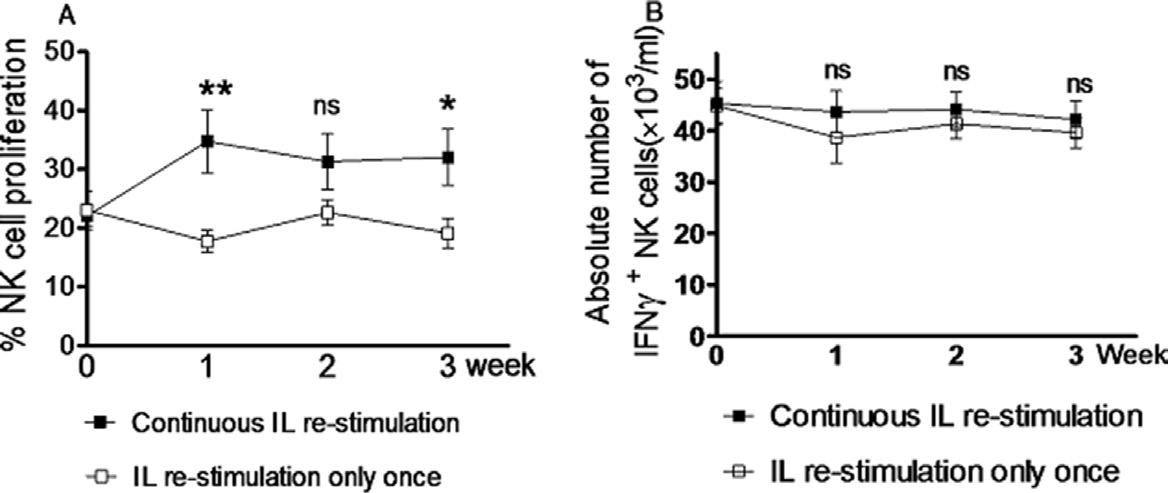
Continuous *in vivo* interleukin re-stimulation is not necessary for NK cells to produce increased amount of IFNγ Rag1KO B6 recipient mice were divided into two groups: the continuous IL re-stimulation group and the IL re-stimulation only once group. Both groups of mice received the same doses of IL-12 (4.5 μg/mouse), IL-15 (5.5 μg/mouse), and IL-18 (22 ng/mouse) for pre-activation. After three weeks, mice further received IL-12 (5 μg/mouse) and IL-15 (8 μg/mouse) for re-stimulation either only once (the IL re-stimulation only once group) or weekly (the continuous IL re-stimulation group). The percentages of NK cells proliferation (**A**) and IFNγ^+^ NK cells (**B**) were determined by flow cytometry every week after IL re-stimulation. (□) the IL re-stimulation only once group; (■) the continuous IL re-stimulation group. Values indicate mean ± SEM (*n* = 12 per group, *p < 0.05, **p > 0.01, and ns = not significant, *p* > 0.05).

To test whether continuous *in vivo* IL re-stimulation is required for generation of memory-like NK cells, the recipient Rag1KO B6 mice were divided into the continuous and discontinuous IL re-stimulation group. Both groups of mice received pre-activation with IL-12, IL-15, and IL-18, after which re-stimulation with IL-12 and IL-15 was given only once to mice in the discontinuous IL re-stimulation group, while mice in the continuous IL stimulation group were administered weekly with the same doses of IL-12 and IL-15. After IL re-stimulation, NK cells harvested from peripheral blood were analyzed by flow cytometry every week. The results revealed that although continuous IL re-stimulation produced more NK cells than IL re-stimulation only once, at first (34.76 ± 5.43% vs 17.78 ± 1.90%, *p* = 0.0074), second (31.34 ± 4.78% vs 22.67 ± 2.18%, *p* = 0.1131), or third week (32.08 ± 4.73% vs 19.15 ± 2.53%, *p* = 0.0247; Figure 2A and Table 1), there was no significant difference in the absolute number of NK cells producing increased IFNγ (IFNγ^+^ NK cells) between two groups at first (43.67 ± 4.05 × 10^3^/ml vs 38.63 ± 4.91×10^3^/ml, *p* = 0.4369), second (44.11 ± 3.45 × 10^3^/ml vs 41.33 ± 2.85 × 10^3^/ml, *p* = 0.5408), or third week (42.18 ± 3.60 × 10^3^/ml vs 39.63 ± 2.97 ×10^3^/ml, *p* = 0.5903; Figure 2B and Table 1). Therefore, these results indicate that although continuous *in vivo* IL re-stimulation promotes the proliferation of NK cells, it is not required for maintaining the levels of NK cells with enhanced IFNγ production. They also suggest that *in vivo* IL re-stimulation only once might be able to induce long-term memory-like of NK cells to produce increased amount of IFNγ, consistent with the previous observation that NK cells after pre-activation by cytokines can keep a stable memory-like state with increased IFNγ production [3].

**Table 1:** Comparison of NK cell kinetics between *in vivo* IL pre-activation with and without re-stimulation

Group	Week 1	Week 2	Week 3
**Syngeneic NK cell transfusion**			
**Donor NK cell proliferation (%) in PB of the recipients**			
1st IL	3.667 ± 0.84%	4.63 ± 0.75%	3.76 ± 0.59%
2nd IL	18.52 ± 1.69%	17.36 ± 2.46%	15.76 ± 2.71%
**Donor IFNγ ^+^ NK cells (%) in PB of the recipients**			
1st IL	5.06 ± 0.79%	4.73 ± 1.09%	4.46 ± 0.97 %
2nd IL	9.53 ± 1.36%	9.03 ± 1.96%	9.86 ± 1.27%
**NK cell proliferation (%) in PB of the recipients**			
Donor NK cells (2nd IL)	21.75 ± 1.56%	20.62 ± 2.64%	20.91 ± 2.16%
Recipient NK cells	5.04 ± 0.92%	4.95 ± 0.99%	5.88 ± 0.80%
**IFNγ ^+^ NK cells (%) in PB of the recipients**			
Donor NK cells (2nd IL)	9.32 ± 0.63%	8.86 ± 0.87%	8.47 ± 0.86%
Recipient NK cells	2.41 ± 0.42%	2.94 ± 0.24%	2.46 ± 0.32%
**NK cells transfusion into MCs**			
**Donor NK cell proliferation (%) in PB of the recipients**			
1st IL	4.25 ± 0.65%	3.68 ± 0.58%	3.23 ± 0.34%
2nd IL	19.64 ± 2.14%	22.94 ± 2.35%	22.61 ± 3.08%
**Donor IFNγ ^+^ NK cells (absolute number/ml, x103) in PB of the recipients**			
1st IL	9.85 ± 1.65	9.35 ± 1.15	8.79 ± 1.41
2nd IL	38.35 ± 6.35	36.05 ± 5.45	33.73 ± 4.23
**NK cells proliferation (%) in PB of the recipients**			
Donor NK cells (2nd IL)	19.64 ± 2.14%	22.94 ± 2.35%	22.61 ± 3.08%
Recipient NK cells	3.65 ± 0.69%	3.85 ± 0.35%	3.38 ± 0.71%
**IFNγ ^+^ NK cells (absolute number/ml, x103) in PB of the recipients**			
Donor NK cells (2nd IL)	38.35 ± 6.35	36.05 ± 5.45	33.73 ± 4.23
Recipient NK cells	10.43 ± 0.93	11.54 ± 2.24	10.08 ± 2.48

1st IL, IL pre-activation only; 2nd IL, IL pre-activation and re-stimulation ; PB, peripheral blood; MCs, a mixed chimera model of the bone marrow transplantation from syngeneic BALB/c plus Rag1KO B6 to BALB/c.

### Memory-like properties of NK cells induced by *in vivo* interleukin pre-activation and re-stimulation can be passed to next generation cells

**Figure 3:**
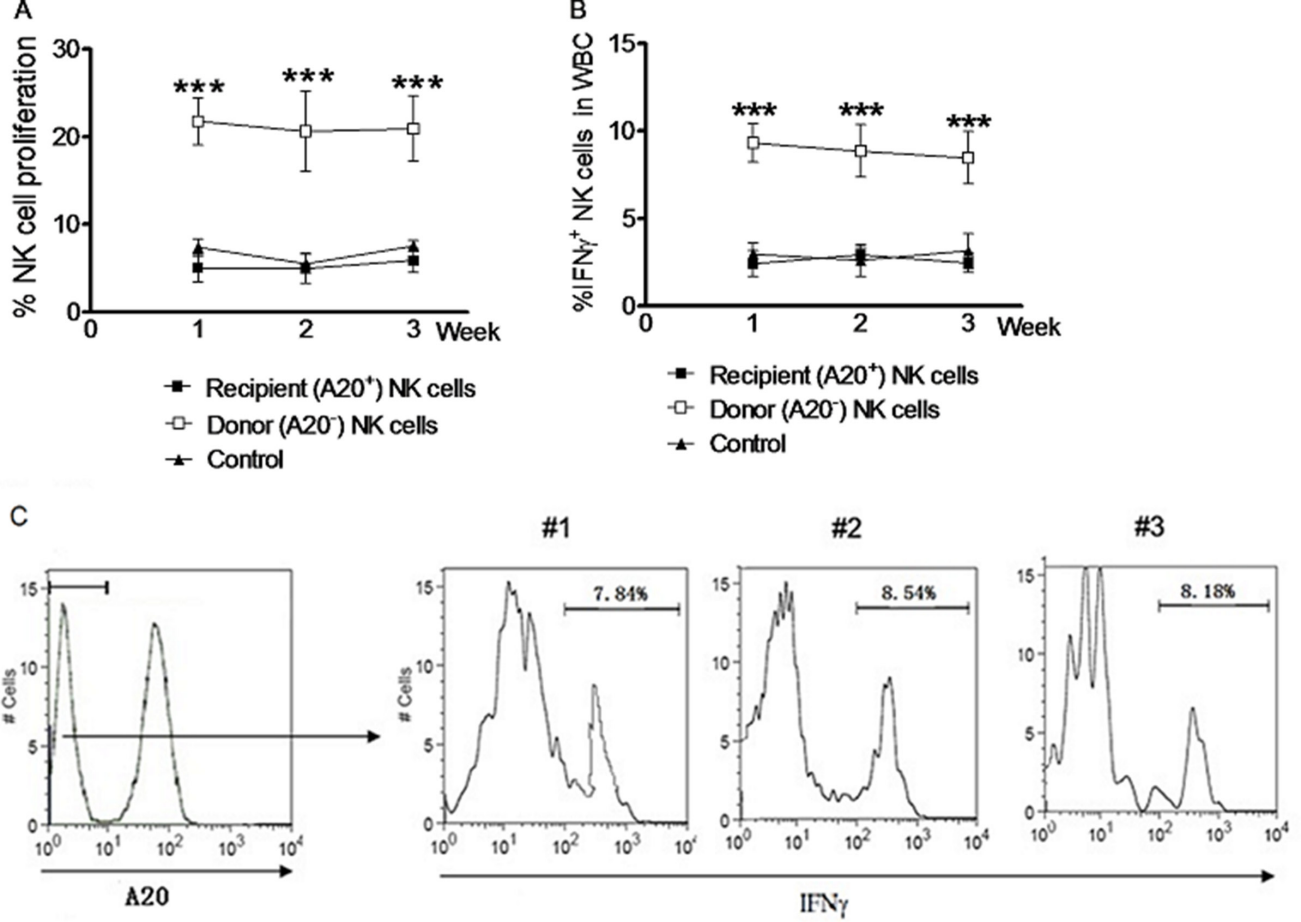
NK cells induced by *in vivo* IL pre-activation and re-stimulation remain the enhanced capacity to produce IFNγ after syngeneic adoptive infusion NK cells from the spleen of Rag1KO B6 (CD45.2+) donor mice were harvested by negative selection after received IL-12 (4.5 μg/mouse), IL-15 (5.5 μg/mouse), and IL-18 (22 ng/mouse) for pre-activation and following IL-12 (5 μg/mouse) and IL-15 (8 μg/mouse) for re-stimulation. Equal numbers (2 × 10^6^) of NK cells were adoptively transferred to syngeneic B6 (CD45.1+) recipient mice. After three weeks, NK cells from peripheral blood of the recipients were harvested and analyzed by flow cytometry. Donor and recipient NK cells were distinguished using anti-A20 antibody. NK cells were labeled with 2.5 mM CFSE before adoptive transfer or flow cytometry analysis. (**A**) The percentages of cell proliferation were determined for A20^−^ donor NK cells (□) and A20^+^ recipient NK cells (■) at the indicated interval after adoptive transfer. (**B**) In parallel, the percentages of IFNγ^+^ NK cells were determined for A20^−^ donor NK cells (□) and A20^+^ recipient NK cells (■) at the indicated interval after adoptive transfer. For panels A and B, the baseline percentage of A20^+^ NK cell proliferation in the recipient mice without adoptive transfer is shown as control (▲). For panels 3A and 3B, values indicate mean ± SEM (*n* = 12 per group, *** *p* < 0.0001). (**C**) After IL pre-activation and re-stimulation as above, IFNγ production in the A20^−^ donor NK cell population was monitored in parental (#1) and daughter (#2, second generation; #3, third generation) NK cells 7 days after adoptive transfer. Different generation memory-like NK cells were identified based on dilution of CFSE labeling. Values indicate the mean of 3 independent experiments (*n* = 15).

We further compared the properties of NK cells induced by *in vivo* IL pre-activation and re-stimulation between the donors and the recipients after NK cell ACT in syngeneic mice. NK cells were harvested through negative selection from the spleen of the Rag1KO B6 (CD45.2+) donors after *in vivo* IL pre-activation and re-stimulation. Equal numbers of these NK cells were adoptively transferred to the syngeneic B6 (CD45.1+) recipients. Within three weeks after ACT, NK cells were harvested every week from peripheral blood of the donors or recipients and labeled with CFSE for further analysis. A20-negative NK cells of the Rag1KO B6 (CD45.2+) donors were distinguished from A20-positive NK cells of the B6 (CD45.1+) recipients by flow cytometry. The proliferation rate of NK cells from peripheral blood of the donors was significantly higher than that of the recipients at first week (21.75 ± 1.56% vs 5.04 ± 0.92%, *p* < 0.0001), second (20.62 ± 2. 64% vs 4.95 ± 0.99%, *p* < 0.0001), or third week (20.91 ± 2.16% vs 5.88 ± 0.80%, *p* < 0.0001; Figure 3A and Table 1). Similarly, the percentage of NK cells producing IFNγ (IFNγ^+^ NK cells) was also significantly higher in the donors than that in the recipients at first (9.32 ± 0.63% vs 2.41 ± 0.42%, *p* < 0.0001), second (8.86 ± 0.87% vs 2.94 ± 0.24%, *p* < 0.0001), or third week (8.47 ± 0.86% vs 2.47 ± 0.32%, *p* < 0.0001) (Figure 3B and Table 1).

We then analyzed whether next generation NK cells derived from the donor NK cells induced by *in vivo* IL pre-activation and re-stimulation could maintain the enhanced capacity to produce IFNγ. To this end, equal numbers of the donor NK cells labeled with CFSE were adoptively transferred to the syngeneic B6 (CD45.1+) recipients. Notably, similar percentages of NK cells with enhanced IFNγ production (IFNγ^+^ NK cells) was observed in the population of A20-negative NK cells, when comparing parental (first generation) and daughter cells (second or third generations) (Figure 3C). Together, these results indicate that the memory-like properties of NK cells with enhanced IFNγ production induced by *in vivo* IL pre-activation and re-stimulation was independent of NK cell proliferation. They also suggest that this enhanced capacity of NK cells to produce IFNγ represents an intrinsic memory-like property of NK cells generated by *in vivo* IL pre-activation and re-stimulation, which thereby could be passed to next generation NK cells.

### NK cells induced by *in vivo* interleukin pre-activation and re-stimulation remain their memory-like properties in an allogeneic bone marrow transplantation (BMT) model

**Figure 4:**
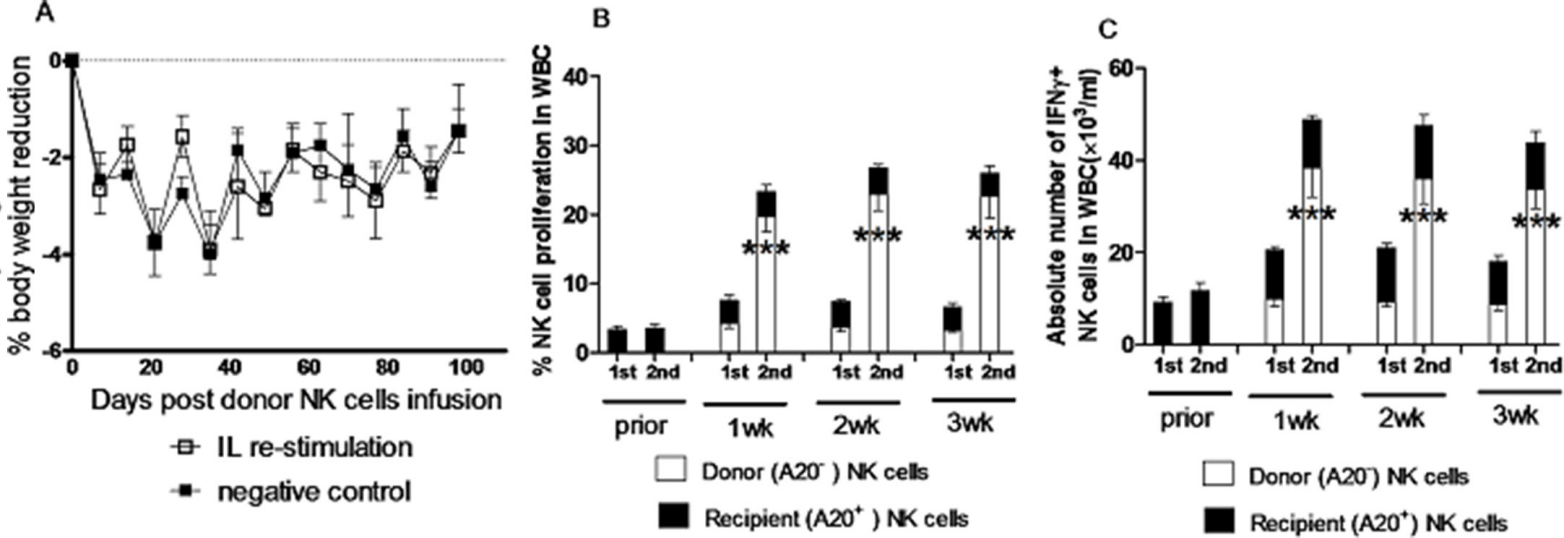
NK cells induced by *in vivo* IL pre-activation and re-stimulation display their enhanced capacity to produce IFNγ, while did not cause GVHD in allogeneic transplantation A mixed chimeras (MCs) mouse model were prepared by injection with a mixture of 0.5 × 10^7^ BMCs from TCD syngeneic BALB/c mice and 1.5 × 10^7^ BMCs from TCD allogeneic Rag1KO B6 mice into lethally irradiated (8 Gy) BALB/c mice. At 8 weeks after injection of TCD BMCs, donor NK cell infusion was performed using NK cells (2 × 10^6^) from the spleen of Rag1KO B6 mice after IL pre-activation (4.5 μg/mouse IL-12, 5.5 μg/mouse IL-15, and 22 ng/mouse IL-18) and/or re-stimulation (5 μg/mouse IL-12 and 8 μg/mouse IL-15). (**A**) The changes of body weight were measured in the pre-activation only group (negative control, ■) and the re-stimulation group (IL re-stimulation, □). Values indicate mean ± SEM (*n* = 12 per group, *p* > 0.05 for each indicated day post donor cell infusion). (**B**) The percentage of NK cell proliferation in white blood cells (WBC) of peripheral blood was determined for the donor and recipient NK cells in the pre-activation only group (1st) and the re-stimulation group (2nd) at the indicated interval after donor NK cell infusion. (**C**) In parallel, the absolute number of donor IFNγ^+^ NK cells was determined in the pre-activation only group (1st) and the re-stimulation group (2nd) at the indicated interval after donor NK cell infusion. For panels 4B and 4C, values indicate mean ± SEM (*n* = 23 per group, ***p < 0.0001 for comparison of donor NK cell proliferation between 1st and 2nd groups).

NK cells, as the first type of cells reconstituted after transplantation, play a pivotal role in controlling infections and leukemia relapse, which still remain the major obstacles of hematopoietic stem cell transplantation. In this context, memory-like NK cells generated by *in vivo* IL pre-activation and re-stimulation were tested in a mixed chimera model for BMT from syngeneic BALB/c plus Rag1KO B6 to BALB/c mice. At eighth week post BMT, the mixed chimeras (MCs) were infused with NK cells harvested from the Rag1KO B6 donor mice that had received IL pre-activation and re-stimulation as described above three weeks prior to transplantation, after which body weight changes were monitored. The BALB/c recipient mice of BMT were divided into two groups: one group of mice received NK cells from the spleen of Rag1KO B6 mice with IL pre-activation only, and another group of mice received the equal number of NK cells from the spleen of Rag1KO B6 mice with both IL pre-activation and re-stimulation. Of note, there was no significant difference in body weight change between these two groups (Figure 4A, *p* > 0.05 for all time points), suggesting that the adoptive transfusion of NK cells induced by *in vivo* IL re-stimulation has no significant effect on graft-versus-host disease (GVHD) of the BMT recipient mice. We further assessed the kinetics of the donor and recipient NK cells labeled with CFSE in peripheral blood of mice after infusion of NK cells. Interestingly, there was also no significant change in the proliferation rate of the recipient NK cells prior to or post donor NK cell infusion in the IL pre-activation only group. However, the proliferation rate of the donor NK cells was significantly increased in the IL re-stimulation group, compared to the pre-activation only group at first (19.64 ± 2.14% vs 4.25 ± 0.65%, *p* < 0.0001), second (22.94 ± 2.35% vs 3.68 ± 0.58%, *p* < 0.0001), and third week (22.61 ± 3.08% vs 3.23 ± 0.34%, *p* < 0.0001) after infusion of the donor NK cells (Figure 4B and Table 1). Similarly, there was also no significant difference in the absolute number of recipient IFNγ^+^ NK cells between prior to or post donor NK cell infusion, while the donor IFNγ^+^ NK cells were significantly increased in the IL re-stimulation group, compared to the pre-activation only group, at first (38.35 ± 6.35×10^3^/ml vs 9.85 ± 1.65 × 10^3^/ml, *p* < 0.0001), second (36.05 ± 5.45×10^3^/ml vs 9.35 ± 1.15 × 10^3^/ml, *p* < 0.0001), and third week (33.73 ± 4.23 × 10^3^/ml vs 8.79 ± 1.41 × 10^3^/ml, *p* < 0.0001) after donor NK cells infusion (Figure 4C and Table 1).

Together, these results indicate that the *in vivo* IL re-stimulation is capable to increase proliferation of NK cells as well as the absolute number of IFNγ^+^ NK cells in this model of BMT. They also suggest that memory-like NK cells with enhanced IFNγ production generated by *in vivo* IL pre-activation followed by re-stimulation might also be used not only in syngeneic mice, but also in allogeneic mice, thereby probably in leukemia patients via allogeneic BMT as well.

### Memory-like NK cells induced by *in vivo* interleukin pre-activation and re-stimulation prolong animal survival in a leukemia xenograft model

**Figure 5:**
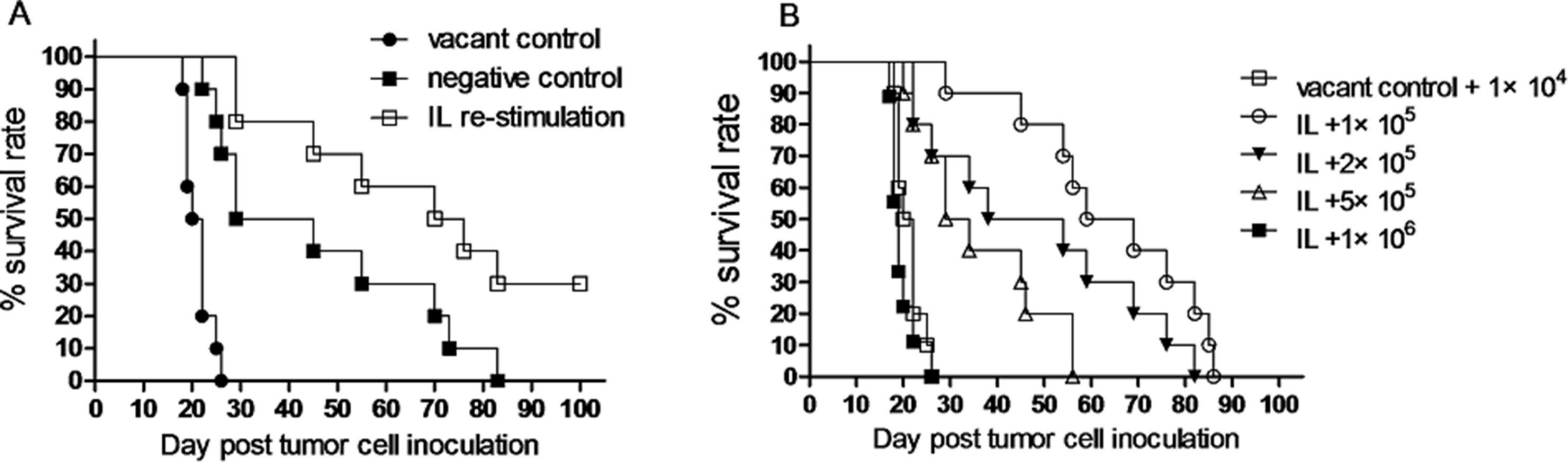
NK cells induced by *in vivo* IL pre-activation and re-stimulation prolongs survival of leukemia mice (**A**) Rag1KO B6 (CD45.2+) mice were divided into three groups: (●) one group of mice received neither IL pre-activation nor re-stimulation as vacant control; (■) one group of mice received IL pre-activation only as negative control; (□) the experimental group of mice received both pre-activation (4.5 μg/mouse IL-12, 5.5 μg/mouse IL-15, and 22 ng/mouse IL-18) and re-stimulation (5 μg/mouse IL-12 and 8 μg/mouse IL-15). After IL stimulation, all mice were inoculated with 1 × 10^4^ Notch1-T-ALL leukemia cells. Kaplan-Meier analysis was performed to assess survival of mice (median survival time: 21 days for the vacant control group, 37 days for the negative control group, and 73 days for the IL re-stimulation group (*n* = 10 per group, *p* < 0.05). (**B**) Mice received both IL pre-activation and re-stimulation as above were inoculated with 1 × 10^5^, 2 × 10^5^, 5 × 10^5^, and 1 × 10^6^ Notch1-T-ALL cells, respectively. Mice received no IL stimulation as described above as vacant control were inoculated with 1 × 10^4^ leukemia cells. Kaplan-Meier analysis was performed to assess survival of mice. Median survival time: 21 days for control (□), 64 days for 1 × 10^5^ cells (○), 46 days for 2 × 10^5^ cells (▼), 31 days for 5 × 10^5^ cells (∆), or 19 days for 1 × 10^6^ cells (■), respectively (*n* = 10 per group, *p* < 0.05 for comparison between 1 − 5 × 10^5^ cells vs. control, *p* > 0.05 for 1 × 10^6^ cells vs. control).

To examine anti-leukemia activity of memory-like NK cells with enhanced IFNγ production induced by *in vivo* IL pre-activation and re-stimulation, Rag1KO B6 (CD45.2+) mice were divided into three groups: the vacant control group, in which mice received PBS; the negative control group, in which mice received IL pre-activation only; and the IL re-stimulation group, in which mice received both IL pre-activation and re-stimulation as described above. After NK cell stimulation, all three groups of mice were inoculated with 1 × 10^4^ Notch1-T-ALL leukemia cells. Of note, while IL pre-activation only had a moderate effect (37 days vs 21 days for vacant control, *p* < 0.05), median survival of animal was significantly prolonged in the IL re-stimulation group (73 days), compared to those in both the vacant control group (21 days, *p* < 0.05) and the negative control group (37 days, *p* < 0.05). Moreover, 30% mice in the IL re-stimulation group had a long-term survival (> 100 days, Figure 5A and Table 2). Furthermore, to determine how potently NK cells induced by *in vivo* IL re-stimulation could kill leukemia cells in this model, mice with IL re-stimulation were divided into four groups, inoculated with different numbers of leukemia cells (1 × 10^5^, 2 × 10^5^, 5 × 10^5^, or 1 × 10^6^), while the vacant control group of mice received only 1 × 10^4^ leukemia cells. Significantly, median survival of animal was significantly prolonged in the IL re-stimulation groups with inoculation of 1 × 10^5^ (64 days), 2 × 10^5^ (46 days), and 5 × 10^5^ (31 days) leukemia cells, compared to that in the vacant control group (21 days, *p* < 0.05 for each case, Figure 5B and Table 2), till the number of leukemia cells reached 1 × 10^6^. Thus, lethal dose of leukemia cells was about 100 times increased (from 1 × 10^4^ to 1 × 10^6^) in mice received NK cells with *in vivo* IL pre-activation and re-stimulation, compared to those receiving PBS. Together, these results support a notion that NK cells induced by *in vivo* IL pre-activation and re-stimulation have a potent anti-leukemia activity *in vivo*, at least in this xenograft model of Notch1-T-ALL.

**Table 2:** Survival of leukemia mice (*n* = 10 per group) after *in vivo* IL pre-activation and re-stimulation

Tumor cell number	Median OS (d)	Death (*n*)	DFS (*n*)
1 × 10^4^	21	10	0
1 × 10^4^ + 1st IL	37	10	0
1 × 10^4^ + 2nd IL	73	7	3
1 × 10^5^ + 2nd IL	64	10	0
2 × 10^5^ + 2nd IL	46	10	0
5 × 10^5^ + 2nd IL	31	10	0
1 × 10^6^ + 2nd IL	19	10	0
**Antibody neutralization**			
1 × 10^4^ + 2nd IL	75	8	2
1 × 10^4^ + 2nd IL + anti-IFNγ	48	10	0
1 × 10^4^ + 2nd IL + anti-NKG2D	59	10	0

1st IL, IL pre-activation only; 2nd IL, IL pre-activation and re-stimulation ; OS, overall survival ; DFS, disease-free survival; d, day after tumor cell inoculation ; *n*, mouse number.

### IFNγ production is crucial for anti-leukemia activity of memory-like NK cells induced by *in vivo* interleukin pre-activation and re-stimulation

**Figure 6:**
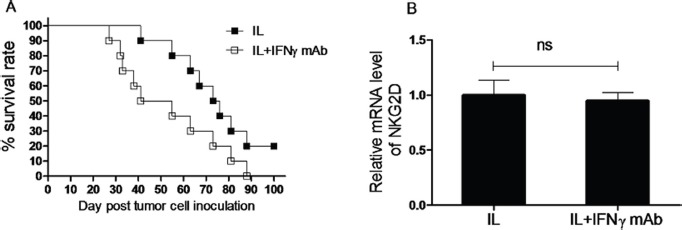
IFNγ production plays an important role in anti-leukemia activity of NK cells induced by *in vivo* IL pre-activation and re-stimulation (**A**) Rag1KO B6 (CD45.2+) mice were divided into two groups: one group of mice received IgG1 as control (IL, ■), and another group of mice received anti-IFNγ antibody for neutralization (IL+INF-g mAb, □). Both groups of mice received pre-activation (4.5 μg/mouse IL-12, 5.5 μg/mouse IL-15, and 22 ng/mouse IL-18) and re-stimulation (5 μg/mouse IL-12 and 8 μg/mouse IL-15) prior to treatment with the antibodies and 1 × 10^4^ Notch1-T-ALL cells post IFNγ neutralization. Kaplan-Meier analysis was performed to assess survival of mice (median survival time: 75 days for the IgG1 control group and 48 days for the anti-IFNγ mAb group. Values indicate mean ± SD (*n* = 10 per group, *p* < 0. 05). (**B**) In parallel, mRNA expression levels of NKG2D were determined by qRT-PCR (*n* = 10 per group, ns = not significant, *p* > 0.05).

To determine whether the enhanced capacity to produce IFNγ is required for the anti-leukemia effect of NK cells induced by *in vivo* IL pre-activation and re-stimulation, anti-IFNγ antibody was employed to neutralize IFNγ secreted by NK cells. To this end, Rag1KO B6 mice were divided into two groups, one group of mice received anti-IFNγ antibody while another group of mice received IgG1 as control for comparison, while IL pre-activation and re-stimulation were given to both groups of mice as described above. After one day, both groups of mice were inoculated with 1 × 10^4^ Notch1-T-ALL leukemia cells. Notably, administration of anti-IFNγ antibody significantly shortened median survival of animal (48 days), compared to IgG1 (75 days, *p* < 0.05, Figure 6A and Table 2). This result suggests that IFNγ production is required for anti-leukemia activity of memory-like NK cells induced by *in vivo* IL pre-activation and re-stimulation.

### NKG2D expression is associated with prolonged survival of leukemia mice after *in vivo* interleukin pre-activation and re-stimulation, likely via promoting IFNγ production

Increased amounts of IFNγ have been shown to decrease NKG2D expression and impair NKG2D-mediated lysis of target cells [19]. We therefore tested whether this mechanism is also applied in this present setting. Rag1KO B6 mice received anti- IFNγ antibody for neutralization or IgG1 as control, followed by IL pre-activation and re-stimulation given to both groups of mice as described above. Two weeks after inoculation of Notch1-T-ALL leukemia cells, mRNA levels of NKG2D were assessed by RT-PCR. As shown in Figure 6B, there was no significant difference in relative mRNA expression of NKG2D between the anti-IFNγ antibody and IgG1 groups (*n* = 10 per group, *p* > 0.05), suggesting that while IFNγ contributes functionally to anti-leukemia activity of NK cells induced by *in vivo* IL pre-activation and re-stimulation, it acts likely via a mechanism independent of NKG2D.

**Figure 7:**
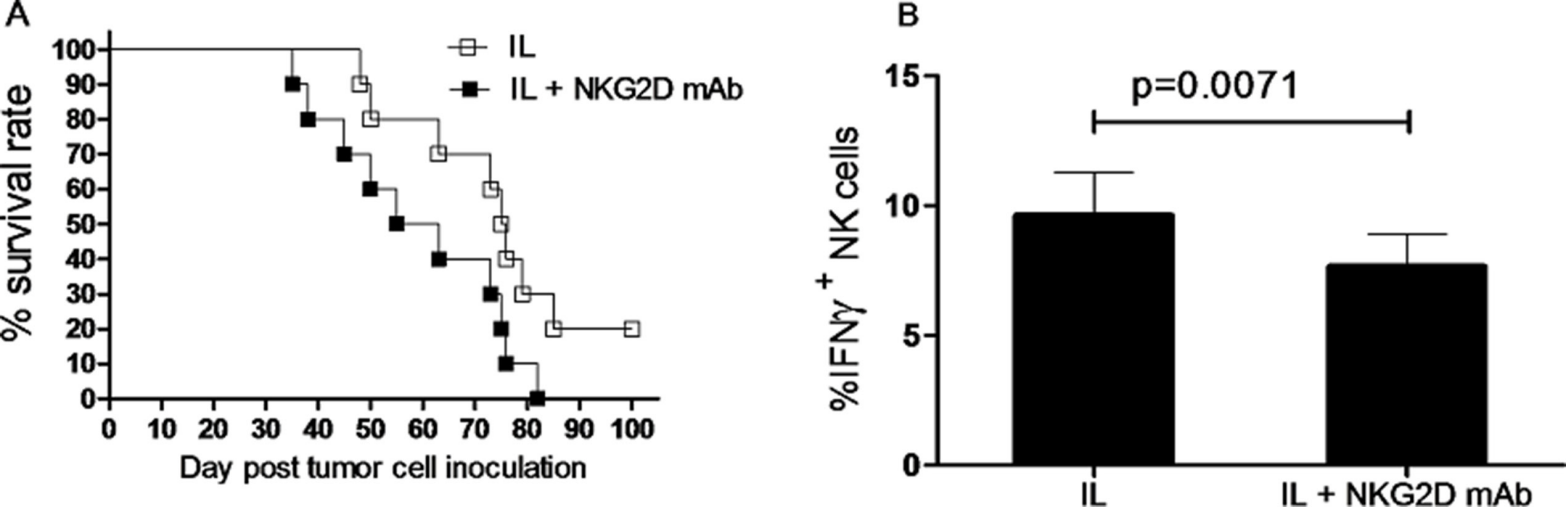
NKG2D expression correlates to survival of leukemia mice after *in vivo* IL pre-activation and re-stimulation (**A**) Rag1KO B6 (CD45.2+) mice were divided into two groups: one group of mice received IgG1 as control (IL, □), and another group of mice received anti-NKG2D antibody for neutralization (IL+NKG2D mAb, ■). Both groups of mice received pre-activation (4.5 μg/mouse IL-12, 5.5 μg/mouse IL-15, and 22 ng/mouse IL-18) and re-stimulation (5 μg/mouse IL-12 and 8 μg/mouse IL-15) prior to treatment with the antibodies and 1×10^4^ Notch1-T-ALL cells post NKG2D neutralization. Kaplan-Meier analysis was performed to assess survival of mice (median survival time: 75 days for the IgG1 control group and 59 days for the anti-NKG2D mAb group. Values indicate mean ± SD (*n* = 10 per group, *p* < 0.05). (**B**) In parallel, mRNA expression levels of IFNγ were determined by qRT-PCR, Values indicate mean ± SD (*n* = 10 per group).

Nevertheless, to test the potential effect of NKG2D expression on survival of leukemia mice receiving IL pre-activation and re-stimulation, Rag1KO B6 mice were divided into two groups, one group of mice received anti-NKG2D antibody for neutralization, another group of mice received IgG1 as control, while both groups of mice were given IL pre-activation and re-stimulation as described above. After one day, both groups of mice were inoculated with 1 × 10^4^ Notch1-T-ALL leukemia cells. Of note, mice with anti-NKG2D antibody neutralization exhibited significant shorter median survival (59 days) than those receiving IgG1 (75 days, *p* < 0.05, Figure 7A and Table 2). To further test whether NKG2D activates or inhibits production of IFNγ, the absolute number of IFNγ^+^ NK cells was analyzed in both the anti-NKG2D antibody neutralization and IgG1 control groups, two weeks after inoculation of Notch1-T-ALL leukemia cells. Unexpectedly, the percentage of IFNγ^+^ NK cells was significantly decreased in the anti-NKG2D antibody neutralization group, compared to the IgG1 control group (9.63 ± 1.65% vs 7.65 ± 1.24%, *p* = 0.0071, Figure 7B). Together, these findings suggest that while IFNγ fails to decrease NKG2D expression, NKG2D levels correlate to prolonged animal survival of mice receiving *in vivo* IL pre-activation and re-stimulation, likely in association with increased IFNγ production.

## DISCUSSION

Although memory-like NK cells with enhanced IFNγ production can be generated by *in vitro* IL re-stimulation [3], they often loss their “effector” function after ACT [16], thereby displaying limited benefit in controlling tumor growth or improving survival. The present study provided evidence supporting that a novel approach of *in vivo* IL pre-activation followed by *in vivo* re-stimulation once was able to induce memory-like properties of NK cells with enhanced IFNγ production, and significantly, these memory-like NK cells retained their enhanced capacity to produce IFNγ in mice for at least three weeks, despite the fact that the half life of NK cells is only about 7 days [18]. An issue then arises whether continuous administration of ILs for re-stimulation, which could cause serious adverse events [20], is required to maintain the memory-like properties of NK cells induced by this approach. Of note, the present results indicated that continuous IL re-stimulation was not necessary for sustaining the absolute number of NK cells with enhanced IFNγ production (i.e., IFNγ^+^ NK cells), though it could increase total number of NK cells. Moreover, it was also observed that memory-like NK cells with enhanced IFNγ production after *in vivo* IL pre-activation and re-stimulation could be passed to their daughter cells with similar properties. It is noteworthy that in addition to ACT in syngeneic mice, NK cells induced by *in vivo* IL pre-activation and re-stimulation could be transfused into a mixed chimera (MC) model of BMT, without effects on the incidence of graft-versus-host disease (GVHD) of the recipient mice, an adverse event recently reported in a study of allogeneic NK cells pre-activated *in vitro* [21]. Furthermore, in this MC model of BMT, the memory-like properties of NK cells to produce IFNγ also persisted for at least three weeks after transfusion. Significantly, NK cells induced by *in vivo* IL pre-activation and re-stimulation significantly prolonged survival of animal in a xenograft model of Notch1-T-ALL, arguing their *in vivo* anti-leukemia activity. Last, the anti-leukemia activity of these memory-like NK cells was associated with increased IFNγ production and expression of the NK cells activation receptor NKG2D which promotes IFNγ production.

Recent approaches for NK cell ACT have been focusing on infusion of NK cells after *ex vivo* activation and expansion with cytokines including IL-2, IL-12, IL-15, IL-18, IL-21, and type I interferons [22]. In this context, it has been reported that NK cells can be pre-activated and expanded by *ex vivo* stimulation with IL-12, IL-15, and IL-18 alone or in combination, which produces the memory-like IFNγ response upon re-stimulation *in vitro* and *in vivo* [5, 23]. However, NK cells pre-activated only by these ILs administered in combination has exhibited promising activity against established tumors in mice, while combined treatment with irradiation is essential for the anti-tumor activity of transferred NK cells [24]. The present findings indicate that *in vivo* IL pre-activation and re-stimulation lead to generation of NK cells with enhanced IFNγ production, but suggest that this new approach resulted in a long-term gain of these memory-like properties in both parental NK cells and their daughter cells which have never been exposed directly to ILs. These results are consistent with the previous finding that NK cells activated by cytokines can differentiate into a stable memory-like state with enhanced IFNγ production upon re-stimulation [5]. However, this enhanced capacity to produce IFNγ is independent of NK cell proliferation *in vitro*, an event distinct from short-term arming/priming during infection [3]. While the memory of some NK cells is driven by antigens, it is unlikely that NK cells could generate antigen-specific memory against a broad range of pathogens, given the fact that they only express the germ line-encoded receptors. However, NK cells do express the receptors for and therefore exquisitely respond to many cytokines, including IL-12, IL-15, and IL-18. For example, after pre-activated overnight *in vitro* with IL-12, IL-18, and IL-15, NK cells have been transferred into the recipient mice. One week after ACT, a higher frequency of NK cells with enhanced IFNγ production upon re-stimulation with IL-2 has been observed, when compared to NK cells without re-stimulation [23], suggesting that re-stimulation with cytokines is able to elicit a robust response *in vivo*. Notably, such an enhanced response induced by *in vitro* re-stimulation also occurs in parental cells that have not undergone division as well as their daughter cells that are the product of division [5]. However, unlike viral antigen-driven memory NK cells, memory-like NK cells induced by *in vitro* IL re-stimulation exhibit neither a distinct cell surface phenotype, nor enhanced cytotoxicity upon re-stimulation [2, 3, 17]. In contrast, the present results suggest that *in vivo* IL pre-activation followed by *in vivo* IL re-stimulation might produce memory-like NK cells with marked cytotoxicity *in vivo* towards tumor cells, at least in the Notch1-T-ALL mouse model.

Whereas allogeneic hematopoietic cell transplantation (allo-HCT) remains a potentially curative treatment for leukemia, its clinical benefit has been limited by high morbidity and mortality of graft-versus-host disease (GVHD). Therefore, the development of a strategy to achieve anti-tumor responses without causing GVHD represents a major challenge in the field of allo-HCT. In a clinical trial, delayed donor lymphocyte infusion (DLI) following establishment of mixed chimerism has been reported to potentially cure hematopoietic malignancies, including leukemia and lymphoma, while a high incidence of GVHD was observed in mixed chimeric patients after DLI [25]. In a mixed chimera model of BMT from syngeneic BALB/c plus Rag1KO B6 to BALB/c, no host-versus-graft reaction has been observed, while the graft-versus-host reaction has occurred [26]. Notably, it has recently reported that transfusion with allogeneic NK cells pre-activated *in vitro* might cause GVHD as severe adverse effect [21]. However, in the present study, we did not observe significant GVHD in the mixed chimera of BMT after transfusion with donor NK cells induced by *in vivo* IL pre-activation and re-stimulation, while NK cells remained the memory-like properties with enhanced IFNγ production for at least three weeks after transfusion. Interestingly, the proliferation rate of NK cells did not significantly decreased in the mixed chimera, compared to that in the model of syngeneic mice, indicating that NK cells might not inhibited by mixed chimerism. Thus, as NK cells induced by *in vivo* IL pre-activation and re-stimulation did not cause GVHD after allogeneic transfusion, this approach might be used in haploidentical bone marrow transplantation. To this end, in two clinical trials [27] designed to test the safety of infusion with T-cell donor lymphocyte enriched with NK cells, 30 patients with high-risk diseases (defined as high risk cytogenetics or those in second remission), who had T-cell depleting non-myeloablative allogeneic stem cell transplant (haploidentical in one study, an average of 9.2 × 10^6^ NK cells were infused; and fully matched in another study, a median of 10.6 × 10^6^ NK cells were infused), received NK-cell enriched donor lymphocyte infusions. It is noteworthy that GVHD was moderate in these patients (approximately 42% in each cohort) and patients survived with remission, after one year follow-up for overall survival. In the present study, 2 × 10^6^ donor NK cells isolated from the spleen of Rag1KO B6 mice were infused in the mixed chimera model of BMT, GVHD did not occur, demonstrating the safety of NK cell transfer as donor lymphocyte infusions (DLI).

The bulk of clinical studies have investigated the use of NK cells to treat a variety of cancers, including hematologic malignancies, particularly acute myeloid leukemia (AML). The first trial of successful ACT and expansion of haploidentical NK cells was reported by Miller and colleagues at the University of Minnesota in 2005 [28]. While transfusion of NK cells did not improve durable response and all patients eventually relapsed, five patients with AML achieved a complete remission (CR) after engrafted with NK cells. A recent study demonstrated that patients with deficient NK cell profiles, including reduced expression of some activating NK receptors (e.g., NKp46 and NKG2D) and decreased IFNγ production, have a significantly increased risk of disease relapse, independently of cytogenetic classification [29]. IFNγ secreted by NK cells has numerous effects on tumor cells, including activation of macrophages, up-regulation of class I expression by antigen presenting cells (APC), polarization of type 1 T-helper cell (Th1), and direct anti-proliferative activity. Moreover, it has also been demonstrated that IFNγ secreted by NK cells is involved in the induction of graft-versus-leukemia (GVL) effects in IFNγ-deficient allo-HCT models, while inhibits GVHD [30, 31]. The present results indicated that IFNγ is functionally involved in the mechanism of action for NK cells induced by *in vivo* IL pre-activation and re-stimulation to kill leukemia cells, consistent with the previous finding that NK cells act against leukemia cells via IFNγ secretion [32]. NK cells can also directly inhibit tumor cell proliferation as well as augment the adaptive immune response against tumor cells via cytokine secretion [33]. To this end, NK cells are able to recognize tumor cells as a target, suggesting a possibility for using NK cell ACT as a cancer therapy [7, 34]. Further, the capacity of NK cells to kill tumor cells likely depends on the combined effects of suppressive and stimulatory signals initiated through cell surface receptors.

One of the key receptors for NK cell activation is NKG2D that has multiple ligands, including MICA, MICB, and ULBP, which are preferentially expressed following cellular stress, infection, or DNA damage [8, 9]. There is the bulk of evidence for an important role of NKG2D in NK cell-mediated antitumor activity *in vitro* and *in vivo* in animal models [35–39]. For example, resting NK cells secrete high levels of IFNγ in response to agonists of TLR3 or TLR7 and IL-12, an effect further enhanced by co-stimulation through NKG2D [13]. Monocytes and NK cells as early source of IFNγ might communicate to each other, via MICA-NKG2D interaction, during an innate immune response to infections in humans [14]. The number of NK cells secreting IFNγ is significantly reduced when NKG2D is blocked by anti-NKG2D antibody [15]. In contrast to the stimulatory effect of NKG2D on IFNγ, high amounts of IFNγ have been shown to decrease NKG2D expression and impair NKG2D-mediated lysis of target cells [19], which might represent a mechanism for the negative feedback of IFNγ in NK cells. However, IFNγ does not significantly decrease expression of NKG2D on cell surface [34], consistent with the present finding that IFNγ failed to decrease NKG2D expression on NK cells. One possibility is that in these studies, IFNγ might not reach such a high level required for inhibition of NKG2D expression. In addition to IFNγ, many other factors may also be involved in determining the amount of NKG2D expressed on cell surface. For example, the expression or availability of DAP10 and DAP12 is required for NKG2D to be expressed on cell surface. Gamma-chain cytokines such as IL-2, IL-7, IL-12, and IL-15 can increase cell surface expression of NKG2D in human and mouse NK cells [40–42]. Moreover, IL-15 signal not only increases NKG2D expression, but also promotes DAP10 expression, as well as phosphorylates this adaptor molecule to prime NKG2D activation [43]. In contrast, some cytokines (e.g., IL-21, TGFb) can act like IFNγ to decrease NKG2D expression [44–46]. In the present setting that NK cells pre-activated and re-stimulated by IL-12 and IL-15, which might influence NKG2D expression [5], IFNγ failed to decrease NKG2D expression. Inversely, inhibition of NKG2D decreased the expression of IFNγ, suggesting that NKG2D might promote IFNγ expression in this model. Nevertheless, it is noteworthy that both IFNγ and NKG2D might be involved in the mechanism of action underlying anti-leukemia activity of NK cells induced by *in vivo* IL pre-activation and re-stimulation.

In summary, the present study provided the bulk of evidence indicating that *in vivo* IL pre-activation and re-stimulation can generate memory-like NK cells with enhanced IFNγ production, which might represent a novel approach for the NK cell ACT therapy to treat cancer like leukemia. Compared to conventionally-used approaches of *in vitro* IL pre-activation and/or re-stimulation, this approach displayed several important advantages contributing to anti-tumor activity of NK cells. First, the memory-like properties of NK cells with enhanced IFNγ production sustain as a long-term response after infusion, without a requirement of continuous IL re-stimulation, which may cause severe adverse events. Second, such properties can be passed to their daughter cells, without loss or reduction of potential therapeutic benefit. Third, transfusion of these NK cells is safe and does not cause GVHD, a major barrier for allo-HCT that represents a potential curing therapy for the treatment of some hematologic malignancies. Last, NK cells induced by *in vivo* IL pre-activation and re-stimulation is highly effective against tumors like leukemia, at least in the Notch1-T-ALL mouse model. Therefore, this novel approach of *in vivo* IL pre-activation and re-stimulation warrants further attention in the development of NK cell ACT therapy for hematologic malignancies, particularly leukemia.

## MATERIALS AND METHODS

### Animals

This study was approved by the Subcommittee on Research Animal Care of the First Bethune Hospital, Jilin University. C57BL/6 (B6)-LY5.2/Cr (H-2b; CD45.1) mice and BALB/c (H-2d; CD45.2) mice were purchased from the Vital River Laboratory Animal Technology Co. Ltd (Beijing, China). B6.129 Rag1 deficient (Rag1KO B6) mice with the B6 background (H-2b; CD45.2) were bred in the animal facility of the Translational Medicine Institution at the First Bethune Hospital. Mice were housed in pathogen-free conditions and used in accordance with institutional guidelines for animal studies. 8 to 12 week old mice were used in all experiments and randomized between cages to avoid bias. 10–15 mice per group mice were used in all experiments except the one for the mixed allogeneic chimeras, in which the number of mice was increased to 23 animals per group to reduce statistical standard error due to a marked decrease of cell counts in the peripheral blood following allogeneic transplantation.

### Preparation of mixed allogeneic chimeras and infusion of donor NK cells

Mixed allogeneic chimeras and administration of DLI was prepared as described previously [26]. Briefly, mixed chimeras (MCs) were established by injection with a mixture of 0.5 × 10^7^ BM cells (BMCs) from T cell-depleted (TCD) syngeneic BALB/c mice and 1.5 × 10^7^ BMCs from TCD allogeneic Rag1KO B6 mice into lethally irradiated (8 Gy) BALB/c mice. TCD BMCs were prepared by depleting CD4^+^ and CD8^+^ cells with anti-CD4 (L3T4) and CD8a (Ly-2) microbeads using the magnetic-activated cell sorter separation system (Miltenyi Biotech, Auburn, CA). Flow cytometry was used to verify completeness of T-cell depletion (< 1.0% remaining cells with the depleted phenotype) in each experiment. Donor NK cells (2 × 10^6^) was infused using cells isolated from the spleen of Rag1KO B6 at 8 and 12 weeks after initial injection with TCD BMCs.

### Antibodies and flow cytometry

Single cell suspensions of splenocytes or peripheral blood were prepared using the standard techniques. Fc receptors were blocked by using 2.4G2 mAb prior to surface staining with the indicated antibodies. The negative selection of NK cells was determined by staining with cell surface monoclonal antibodies (mAbs), including anti-CD19, anti-CD4, anti-CD8a, anti-CD5, anti-Gr1, and anti-Ter-119 (Miltenyi Biotech). The surface phenotype of NK cells was determined by staining with mAbs, including anti-CD3-PE and anti-NK1.1-APC (Biolegend, San Diego, CA). Intracellular IFNγ was measured by pacific blue-conjugated anti-IFNγ (Biolegend) as described previously [47]. Dead cells were excluded by propidium iodine (PI) staining and live cells were gated on PI-negative cells. The NK cells of donors or recipients were distinguished by using anti-CD45.1 mAb (A20, BD Biosciences, San Jose, CA), FITC-34-2-12 (H-2D^d^ mAb, BD Biosciences), or Bio-KH95 (anti-H-2D^b^ mAb, BD Biosciences). Data acquisition was performed with FACS Calibur flow cytometer (BD Pharmingen, San Jose, CA) using a CellQuest software (BD Biosciences). Data analysis was performed using a FlowJo software [48].

### Cytokine stimulation

Cytokines used in the *in vivo* experiments were reconstituted and dosing as per manufacturer's instructions and given via tail vein in a total volume of 0.2 ml per mouse. In the experiments of NK cells with *in vivo* IL re-stimulation, the combination of IL-12 (4.5 μg per mouse, PeproTech, Rocky Hill, NJ) and IL-15 (5.5 μg per mouse, PeproTech) plus IL-18 (22 ng per mouse, Medical and Biological Laboratories Co., Nagano, Aichi, Japan) was injected into Rag1KO B6 mice as pre-activation. After three weeks, IL-12 (5 μg per mouse) and IL-15 (8 μg per mouse) were given to Rag1KO B6 mice as IL re-stimulation for overnight. In some experiments for testing cell division and proliferation, 2 × 10^6^ NK cells were labeled with 2.5 μM carboxyfluorescein diacetate succinimidyl ester (CFSE, Invitrogen, Carlsbad, CA) and equal numbers of CFSE-labeled NK cells were then used for further experiments. The percentages of NK cells or IFN ©^+^ NK cells harvested from spleen or peripheral blood of mice were determined by flow cytometry.

In some experiments, NK cells from the spleen of Rag1KO B6 (CD45.2+) mice receiving IL pre-activation or re-stimulation were harvested by negative selection using a mixture of antibodies including anti-CD5, anti-Gr1, and anti-Ter-119. Purity of NK cells (90–98%) was determined by flow cytometry with NK1.1 staining. Equal numbers of NK cells were adoptively transferred to syngeneic B6 (CD45.1+). After three weeks, NK cells from peripheral blood of B6 (CD45.1+) were harvested and anti-CD45.1 (A20) antibody was used to distinguish donor NK cells from recipient NK cells. In the experiments for continuous IL re-stimulation, IL-12 (5 μg per mouse) and IL-15 (8 μg per mouse) were given every week post pre-activation with IL-12, IL-15, and IL-18, after which NK cells were analyzed by flow cytometry at the indicated intervals.

In the experiments for NK cells with *in vitro* re-stimulation by cytokines, NK cells from the spleen of Rag1KO B6 (CD45.2+) mice were pre-activated with IL-12 (10 ng/mL), IL-18 (50 ng/mL) and IL-15 (10 ng/mL) overnight as described previously [3]. In next day, equal numbers of NK cells (2 × 10^6^) were adoptively transferred into syngeneic B6 (CD45.1+) mice. After three weeks, enriched NK cells from spleen were harvested by negative selection as described previously [3] and then *in vitro* re-stimulated with IL-12 (10 ng/mL) and IL-15 (100 ng/mL) for 4 hrs, followed by flow cytometric analysis of NK cells in next day.

### NKG2D and IFNγ neutralization studies

Anti-mouse NKG2D mAb (HMG2D) and rat anti-mouse IFN © mAb (Clone XMG1.2) were purchased from BioXCell (West Lebanon, NH). Mice were treated with 250 μg anti-NKG2D mAb (HMG2D) twice weekly [49] or 0.4 μg anti-mouse IFN © mAb (Clone XMG1.2) every other day [50], until the recipients died from leukemia. Mice were treated with IgG1 antibody as control. Flow cytometry using APC-NKG2D or APC-IFN © staining was performed to verify completeness of NKG2D or IFN © depletion (< 1% remaining cells with the depleted phenotype).

### Quantitative RT–PCR

Total RNA was extracted from 1 × 10^7^ cells from the spleen of Rag1KO B6 mice (*n* = 10) 2 weeks after inoculation of leukemia cells, using the Trizol reagent (Invitrogen) as per manufacturer's instructions as described previously [51]. Yield and quality of RNA were assessed using a NanoDrop 1000 spectrophotometer (Thermo Fisher Scientific, Waltham, MA). 1.0 μg of total RNA was reversely transcribed into cDNA using a qScript kit (Quanta Biosciences, Gaithersburg, MD) as per manufacturer's instructions. mRNA levels of the target genes were quantified by Lightcycler II (Roche, Basel, Switzerland) using SYBR II qPCR mixture (Invitrogen). Primers were designed for an anneal temperature of 61°C using a Primer 3 software [52] and synthesized by TAG Copenhagen (Frederiksberg, Denmark). Purified and diluted sequence-verified PCR products (GATC Biotech, Konstanz, Germany) were used to create a standard curve for each primer pair. Expression levels of the target genes were calculated as absolute quantification according to the relevant standard curve, using a Lightcycler Software version 3.53, and then normalized to the housekeeping gene β-actin. Cycling parameters were set up to obtain similar reaction efficiencies between 1.9 and 2.0. The parameters for qPCR cycling were: initial denaturation at 95°C, followed by 45 cycles of 10 second denaturation at 95°C, 5 second anneal at primer-specific temperature (56–60°C) and 15 second extension at 72°C. Following PCR, a melting curve analysis was conducted. Any reactions with CP value > 40 or with a nonspecific peak in the melting curve analysis were considered as negative reactions and arbitrarily given the value of 0 for further statistical analyses.

### Statistical analysis

Student's *t* test was performed to compare the variables between 2 groups, with *P* < 0.05 considered as statistically significant. The data are presented as mean ± SD or mean ± SEM. All flow cytometric analyses and proliferation assays were analyzed using a FlowJo software. RT-PCR data was analyzed using a SSPS software. Survival rate analysis was performed with a Prims4 software.
